# Airinemes: thin cellular protrusions mediate long-distance signalling guided by macrophages

**DOI:** 10.1098/rsob.200039

**Published:** 2020-08-19

**Authors:** Dae Seok Eom

**Affiliations:** Department of Developmental and Cell Biology, University of California, Irvine, CA 92697, USA

**Keywords:** airinemes, cytonemes, cell–cell communication, zebrafish, macrophage, filopodia

## Abstract

Understanding the mechanisms of cell-to-cell communication is one of the fundamental questions in biology and medicine. In particular, long-range signalling where cells communicate over several cell diameters is vital during development and homeostasis. The major morphogens, their receptors and intracellular signalling cascades have largely been identified; however, there is a gap in our knowledge of how such signalling factors are propagated over a long distance. In addition to the diffusion-based propagation model, new modalities of disseminating signalling molecules have been identified. It has been shown that cells can communicate with direct contact through long, thin cellular protrusions between signal sending and receiving cells at a distance. Recent studies have revealed a type of cellular protrusion termed ‘airinemes’ in zebrafish pigment cell types. They share similarities with previously reported cellular protrusions; however, they also exhibit distinct morphology and features. Airinemes are indispensable for pigment pattern development by mediating long-distance Delta-Notch signalling between different pigment cell types. Notably, airineme-mediated signalling is dependent on skin-resident macrophages. Key findings of airineme-mediated intercellular signalling in pattern development, their interplay with macrophages and their implications for the understanding of cellular protrusion-mediated intercellular communication will be discussed.

## 1. Introduction

Cell-to-cell signalling is essential in all multicellular organisms. In particular, paracrine signalling, which enables cells to communicate over several cell diameters, is vital in development and homeostasis. If such signals are deployed at the wrong time or place, they lead to defects, including cancers [1]. Still, we only have limited information about the mechanisms of how signalling molecules are propagated through tissues. The traditional textbook model postulating that signalling molecules propagate between cells via diffusion in the extracellular space is about 60 years old [2–4]. However, it has not been fully explained how cells can communicate precisely and reliably through diffusion-based mechanisms [2–8]. In addition to the diffusion-based signal propagation model, many research groups recently have shown that cells can communicate over substantial distances via direct contact through long, thin cellular protrusions. They resemble typical filopodia but have functions to transmit major morphogenetic signals, and such cellular protrusion-mediated communication has now been observed in various organisms and tissues *in vivo* with functional validations [4,9–17]. Many of these signal-carrying protrusions are orders of magnitude longer than typical filopodia and can extend or retract in a highly dynamic fashion [11,18,19]. While they can differ in their morphology and exact signalling mechanism, all of them function in mediating long-range intercellular communication.

At present, two categories of signal-carrying cellular protrusions have been largely identified: signalling filopodia, also known as cytonemes and tunnelling nanotubes, also known as intercellular bridges [19,20]. However, emerging evidence for such cellular protrusions with distinct features and morphology has been reported recently in various species and contexts. For example, it has been suggested that large vesicle-like structures called migrasomes at the tips of retraction fibre from the rear of the migrating cells are used for long-distance cell–cell communication during Kupffer's vesicle formation in gastrulating zebrafish [21–23]. Such findings suggest that there might be many unidentified forms of cellular protrusions in nature.

The idea that cellular protrusions may function for intercellular communication was suggested as early as the 1960s. Gustafson and Wolpert [24] observed cellular protrusions in developing sea urchin embryos. Similarly, filopodia-like ‘feet’ were seen in developing butterflies in the 1980s [25]. Definitive studies about signalling filopodia were first published in 1995. During gastrulation in sea urchin embryos, primary mesenchymal cells and ectodermal cells extend long thin ‘non-conventional filopodia’, and Miller *et al*. [13] suggested that primary mesenchymal cells seem to acquire positional information not by diffusion but via these cellular protrusions. Several years later, the Kornberg group discovered similar cellular protrusions they named ‘cytonemes’ in *Drosophila* wing imaginal discs [14]. It has been known that Decapentaplegic (Dpp), a bone morphogenetic protein (BMP) homologue, is produced from the signalling centre at the anteroposterior boundary of the disc [26,27]. The Kornberg group found that signal-receiving cells in the anterior and posterior compartments extend cytonemes that contact the Dpp-producing cells at the border. Dpp receptors in the signal-receiving cells move along the cytonemes in a proximal to distal direction from the cell body toward the signal source [14]. Signal transduction, therefore, initiates at the tip of the cytonemes where they contact the Dpp-producing cells. Although it is not well understood how the signalling is triggered at the interface between the cytonemes' tip and the receiving cell surface, cytoneme-mediated signalling was found to be vital for wing disc patterning [28]. Cytonemes are actin-rich cellular protrusions, and also mediate several other major signalling factors, including Fibroblast growth factor (Fgf), Hedgehog (Hh) and Wingless (Wg) in different cell types in *Drosophila* [11,12,28–32].

Cytonemes have been described in vertebrates as well. For example, a Sonic hedgehog (Shh) concentration gradient is required for limb bud development in the chick. Cytonemes extend from both Shh-expressing and -receiving mesenchymal cells in this context, and Shh ligands and receptors localize to the distal ends of cytonemes on these cells, respectively. Thus, the signalling event takes place at the tips of cytonemes [15]. In zebrafish, cytonemes in the neural plate deliver an essential Wnt signal during gastrulation [16,33,34], and bidirectional cellular protrusion-mediated Eph/Ephrin signalling between liver and lateral plate mesodermal cell to coordinate tissue movements [35].

Both diffusible morphogens as well as membrane-bound signals can be transmitted over long distances via cytonemes [9,30,36]. In the *Drosophila* thorax, sensory organ precursors (SOPs) extend Delta-carrying cytonemes that inhibit fate change in cells over several cell diameters away [30,36].

Another kind of cellular protrusions termed, ‘tunnelling nanotubes (TNTs)’, has been described in mammalian cell lines and various species. TNTs are conduit-like projections that allow the transfer of soluble cytoplasmic components, intracellular vesicles and even cellular organelles from signal-sending to -receiving cells. They also have been implicated in their roles in the pathogenesis of diseases [20,37–39].

Recent studies have added complexity to the current knowledge of cellular protrusion-mediated signalling [9,40]. Studies have identified a type of cellular protrusion that transmits a Delta-Notch signal between pigment cells at a distance in zebrafish [9]. These cellular protrusions are called ‘airinemes’ and exhibit many similarities and differences with cytonemes and TNTs. One of the striking differences between airinemes and others is that airineme-mediated signalling relies on skin-resident macrophages, which will be discussed in §3. Macrophages are immune phagocytes that clear dead cells and foreign pathogens [41]. Their novel role in airineme-mediated signalling demonstrates a previously unappreciated function of macrophages in cellular protrusion-mediated signalling between non-immune cells [42]. It is noted, however, that airinemes are reported only in pigment cell types in zebrafish to date. Thus, it is an open question whether or not the airineme-mediated signalling is a general mechanism.

Many reviews have discussed the similarities and differences between known signalling cellular protrusions [12,17–19,36]. This article instead will focus on the details of airineme-mediated signalling between pigment cell types and their dependency on macrophages.

## 2. What are airinemes?

**Figure 1.**
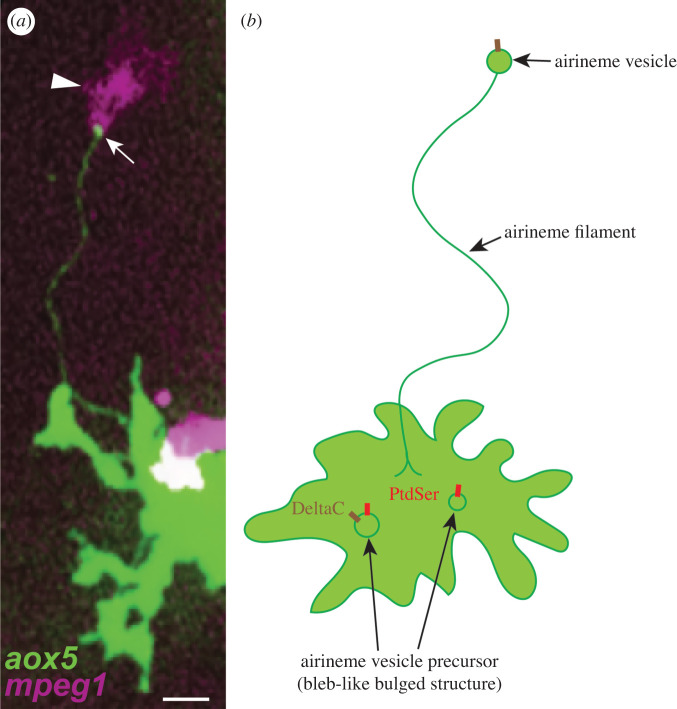
Airinemes and their interaction with macrophages. Airineme by zebrafish aox5+ xanthoblast with membraneous vesicle (white arrow) and pulled by a macrophage (white arrowhead) (*a*). Airinemes possess vesicles at the tip of their filaments. Signalling molecule (DeltaC) containing airineme vesicles originates from airineme vesicle precursors, which appear to be bleb-like structures at the plasma membrane. They are PtdSer-rich and recognized by skin-resident macrophages (*a*, arrowhead and *b*). *The size of the vesicle and its precursors in the cartoon are exaggerated for ease of viewing (*b*). Scale bar: 10 µm (*a*).

Airinemes are long, thin cellular protrusions identified from pigment cells in zebrafish skin. These protrusions mediate long-distance signalling between different pigment cell types during pigment pattern formation. Like other signalling cellular protrusions, airinemes can be visualized with membrane-tethered fluorescence tags [9,14–16]. These are less than a micrometre in diameter, extend up to several hundred micrometers, and dynamically extend and retract. Interestingly, airinemes exhibit long, complex, meandering trajectories and possess a membranous vesicle at their tip (figure 1). Considering these newly identified features and to distinguish these from previously reported signalling cellular protrusions, these are called—‘airinemes’, named after Iris, the messenger of the Gods in Greek mythology, and Sir George Airy, who described the limits of optical resolution [9].

### 2.1. Airineme composition

Most of the cytonemes found in *Drosophila* are actin-based [19]. However, those found in higher animals tend to have both actin and microtubules, but still, tubulin was detected at the base of the cytonemes [19]. Entire airineme filaments and the vesicles are labelled with actin markers such as LifeAct and Calponin homology domain of utrophin (UtrCH). Airineme extension is inhibited by blebbistatin (myosin II inhibitor) or ML141 (cdc42 inhibitor) treatment [9]. Like the cytonemes, airineme extension depends on Cdc42 activity, suggesting that airinemes share some similarities with cytonemes. Since Cdc42 is known to control the cytoskeletal organization and its inhibition potentially blocks other filopodial extensions, it was tested under the condition where low enough induction of dominant-negative Cdc42 affected airineme extension but did not significantly impact regular short filopodia and other protrusions with the cell type-specific and temporally inducible transgenic line [9]. Staining of tubulin alone or in a combination of a membrane-targeted fluorophore, and transient accumulation of microtubule plus-end binding protein EB3 along the airinemes suggest microtubules are components of airinemes as well [9,42]. Consistent with this, nocodazole (tubulin polymerization inhibitor) treatment blocked airineme extension. Thus, it is highly likely airinemes contain actin filaments and microtubules [9]. Airineme vesicles are inconsistently labelled with tubulin markers suggesting dynamic cytoskeletal regulation occurs differentially in airineme filaments and the vesicles, and it remains to be addressed [9].

### 2.2. Airineme vesicles

**Figure 2.**
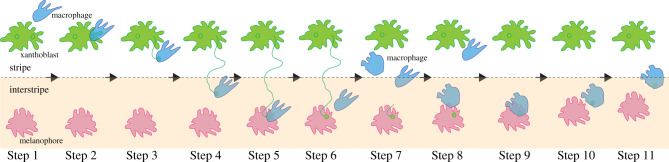
Macrophage-dependent airineme signalling during pigment pattern formation in zebrafish. Step 1: a macrophage approaches a xanthoblast which has airineme vesicle precursors (=bleb-like structure at the plasma membrane, green circles). Step 2: the macrophage recognizes PtdSer-rich airineme vesicle precursor, Step 3: macrophage nibbles and pulls the precursor (=airineme vesicle). Step 4: the airineme filament elongates as macrophage migrates. Step 5: macrophage recognizes target melanophore and unloads the vesicle. Step 6: the macrophage leaves the target, but the airineme vesicle is stabilized at the surface and activates Notch signalling on the target cell surface. Step 7: the airineme filament retracts but the vesicle is still stabilized on the target cell surface for more than an hour. Step 8: another macrophage approaches the stabilized airineme vesicle on the target cell surface. Step 9: this macrophage engulfs the airineme vesicle. Step 10: the macrophage moves away from the target cell and starts to phagocytose the vesicle. Step 11: the engulfed airineme vesicle is finally degraded. After Notch activation at Step 7, the target melanophore migrates toward the stripes from the interstripe.

One of airinemes’ characteristic features is that they possess vesicle-like membranous structures at their tips, and these structures contain DeltaC (and possibly other Delta ligands). Live imaging suggests that airineme vesicles originate from the surface of xanthoblasts, which are the airineme extending undifferentiated/unpigmented yellow pigment cell type in zebrafish (figures 1*b* and 2 Step 1–3) [9]. These airineme vesicles are relayed from the signal sending cells to the target cells by macrophages, which will be discussed further in the next section. Although more detailed and extensive studies are required, airineme vesicle precursors are presumed to be outwardly bulged bleb-like structures and pre-formed at the plasma membrane before the airineme extension (figure 1*b*). These airineme vesicle precursors are abundant in phosphatidylserine (PtdSer), a well-characterized ‘eat-me’ signal for macrophages [41]. They are most frequently observed in airineme producing xanthoblasts but less in differentiated xanthophores, and that correlates with high airineme extension frequency seen in xanthoblasts versus low in xanthophores [9,42]. The underlying molecular mechanisms of how such structures are regulated is not known. Similar outward plasma membrane extrusion can be found in budding-yeast or microvesicles called ‘ectosomes,’ suggesting it might share the same molecular pathways for the formation of the precursors [43]. Another interesting question would be how DeltaC is packaged into airineme vesicle precursors (=airineme vesicles). It seems DeltaC is packaged in the precursors before they are picked up by macrophages but not after or while the airinemes are extending. DeltaC expression is already evident in the airineme vesicle precursors from the surface of xanthoblasts [9]. Interestingly, however, not all such airineme vesicle precursors are DeltaC positives suggesting that they are packaged presumably during maturation of the precursors [9].

## 3. Macrophages in airineme signalling

Airinemes extend up to approximately 250 µm in length and exhibit meandering trajectories, raising the question of how airinemes can reach their target cells that are several cell diameters away across densely packed heterogeneous cell types. Do they autonomously extend, search and reach their targets? Or are there some other mechanisms that guide airinemes? Indeed, it has been revealed that airineme-mediated signalling in zebrafish skin relies on skin-resident macrophages—innate immune cells that scavenge and clear dead cells and foreign pathogens (figure 2) [29,31,37,38]. Ninety-four per cent of airineme extensions have been observed to be associated with macrophages. Also, airineme extensions were severely inhibited when skin-resident macrophages were ablated [42]. Moreover, pigment pattern defects after macrophage depletion mimics the phenotypes shown when airineme extension is inhibited by xanthophore-lineage specific dominant-negative cdc42 expression [9,42]. Overall, this suggests macrophages play an essential role in airineme-mediated intercellular signalling.

As mentioned above, it has been shown that airineme vesicles originate from bleb-like airineme vesicle precursors at the surface of xanthoblasts (figure 1*b*). They are PtdSer positive, the evolutionarily conserved ‘eat-me’ signal for macrophages [29,31,37,39]. Macrophages engulf and pull PtdSer+ precursors/vesicles from the surface of the xanthoblasts, ‘drag’ them as they migrate through the tissue with filaments trailing back to source xanthoblasts, and then deposit them onto the membrane surface of target melanophores. Thus, meandering airineme trajectories reflect the migratory paths of airineme vesicle-bearing macrophages (figure 1*a* and 2). Once deposited, airineme vesicles adhere to target melanophores and stabilize from 1 to 12 h, and the trailing filaments are detached from the vesicles and retracted; presumably, DeltaC ligands at the membrane of the airineme vesicles interact with Notch receptors at the target cell surface and activate Notch signalling during this event. However, there is no evidence whether DeltaC from the vesicle is the ligand for target melanophore Notch activation. It could be activated by other unknown ligands in the vesicle. Also, it is conceivable that the robustness of signalling could be regulated by changing the duration of the vesicle stay/stabilization on the target cells. However, this requires further research. Next, then how are such stabilized airineme vesicles on target cells eliminated? One possible scenario would be the target cells endocytose the vesicles. It has not been observed though that the airineme vesicle fuses into the target cell membrane; instead, other macrophages approach, engulf and they seem to phagocytose the stabilized airineme vesicles from the target cell membrane. Time-lapse movies show that the fluorescence intensity of the labelled airineme vesicles that are completely engulfed by the macrophages rapidly diminishes [8,29] (figure 2, Step 10–11). Thus, these observations suggest that macrophages play critical roles in the initiation and presumably the termination of airineme-mediated long-distance Delta-Notch signalling [29].

There are many remaining questions about the macrophage dependency of airineme-mediated signalling. For example, how airineme vesicles survive from phagocytosis while being dragged by macrophages? In other words, what is the difference between when the vesicles are relayed to the target cells and are eliminated by macrophages after stabilization on the target cell membrane? When macrophages engulf and pull the vesicles, airineme filaments are still connected to the vesicles as previously mentioned (figure 2). Thus, it is conceivable that due to the tethered airineme filaments, macrophages incompletely engulf (nibble) the airineme vesicles but are not able to internalize (swallow) the vesicles. Indeed, airineme vesicles are phagocytosed by macrophages whenever the vesicles are clipped/detached from the filaments. This is often seen when airineme vesicle-bearing macrophages encounter non-target cells [9]. Experiments with the strategies to disconnect the airineme filaments from the vesicles, similar to the axonal severing by high-power laser, would be useful in proving this hypothesis. Another possibility would be that the dynamic regulation of some molecules that prevent the phagocytosis such as CD47 or CD24 at the airineme vesicles [44–46].

Macrophages relay airineme vesicles in a target-specific manner (see §4). Thus, a question is how macrophages or airinemes recognize their targets. It seems the macrophages engulf most of the vesicles except the tethered filaments. Therefore, one of the hypotheses would be that the airineme vesicles deliver an instructive signal to the vesicle engulfed macrophages for the target recognition. It would be interesting to investigate whether macrophage behaviours such as directionality, migration speed or cell morphology are altered before and after they interact with airineme vesicles. Alternatively, macrophages might dynamically expose the incompletely engulfed airineme vesicles while dragging them to probe the environment. Live imaging with super optical- and time-resolution will be essential to prove this hypothesis.

In addition, macrophages' non-immune function in intercellular signalling raises an interesting question: whether there are macrophage subpopulations, and if they are specifically involved in the airineme-mediated signalling. Tissue-resident macrophages are known to be highly heterogeneous, and mpeg1+ ectoderm-derived macrophage-like cells called metaphocytes are identified recently in zebrafish epidermis [41,47]. However, it remains to be determined whether the metaphocytes or other macrophage subpopulations play roles in airineme signalling, or conventional macrophages can perform both signalling and immune function.

Lastly, it has not been reported whether other signalling cellular protrusions are macrophage-dependent or require other cell types for their signalling function. At least, this discovery would raise the possibility that cellular protrusion-mediated signalling consists of not only the signal-sending and -receiving cells but also other intermediate cellular players. Future studies are necessary to determine whether macrophages or other intermediate cell types play similar supporting roles in other types of long-distance signalling.

## 4. Airinemes in pattern formation

**Figure 3.**
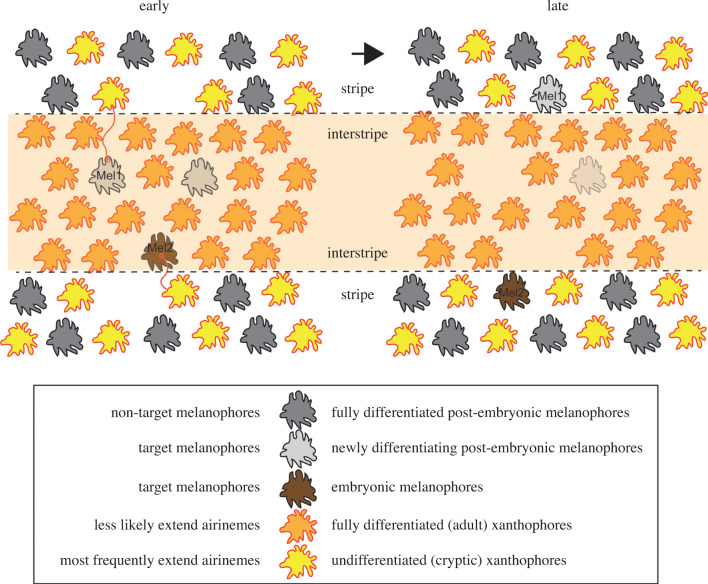
Airineme signalling in pigment pattern development. Airinemes extended from xanthoblasts in stripes stabilized onto newly differentiating melanophores (Mel1) or embryonic melanophore (Mel2) in the interstripe region, and later these two target melanophores consolidated into the stripes.

Adult zebrafish have alternating dark stripes and orange/yellow interstripes. Stripes are composed of dark pigment cells called melanophores and unpigmented yellow xanthoblasts. These unpigmented yellow xanthoblasts are also referred to as ‘cryptic xanthophores’ [48]. Interstripes include differentiated yellow/orange xanthophores (figure 3). The third pigment cell type, iridescent iridophores, is all over the flank. Zebrafish stripe pattern formation is a result of cell–cell interactions between all three pigment cell lineages [49,50]. The most well-studied cell–cell interactions are between xanthophore- and melanophore-lineages. Laser or genetic ablation of either cell type results in disruption of the pigment pattern, and suggests that the interaction between these two cell types are critical for stripe pattern formation [50,51]. Earlier in development, these two cell lineages are intermingled with each other. Some embryonic melanophores develop within the prospective interstripe and stay until metamorphosis (larval-to-adult transition). Also, during this period, some of the post-embryonic melanophores are differentiated within the future interstripe [50]. Repeated daily time-lapse observations revealed that those two melanophore subpopulations are gradually cleared out from the interstripe by coalescing into nearby stripes or cell death. The underlying cellular and molecular mechanisms of interactions between those two cell lineages are not fully understood, but it is thought that the diffusible factors from xanthophores repel melanophores from the interstripe to stripes [3,52].

It has been suggested that airineme-mediated signalling between xanthophore- and melanophore lineages plays an essential role in stripe pattern formation, and that this signalling is dependent on skin-resident macrophages [9,42]. Airineme extension is most frequently observed during zebrafish metamorphosis, and in this developmental stage, various tissue remodelling occurs, including pigment pattern formation [9,50,51,53,54]. Also, airinemes are most frequently extended by undifferentiated/unpigmented xanthoblasts, which are located outside the interstripe but alongside with other fully differentiated melanophores in stripes (figures 2 and 3). The directionality of airineme extensions does not seem significantly biased in any direction (unpublished). However, airinemes stabilized preferentially on newly differentiating melanophores or embryonic melanophores, which are intermingled with xanthophores in the interstripe during metamorphosis [9]. Macrophages relay the DeltaC containing airineme vesicles to those two types of target melanophores which in turn, activates Notch signalling. Notch activation in target melanophores may activate the downstream signalling pathway required for melanophore migration and survival [55,56]. Inhibition of airineme extension significantly decreased the number of Notch activated melanophores, which results in pigment pattern failure [9]. Since airineme extension relies on macrophages, macrophage ablation leads to the inhibition of airineme extension; this results in the failure of pigment pattern formation [42]. Thus, macrophage/airineme-mediated long-range signalling between pigment cell types are critical for proper pigment patterning.

## 5. Airinemes in various cell types

**Figure 4.**
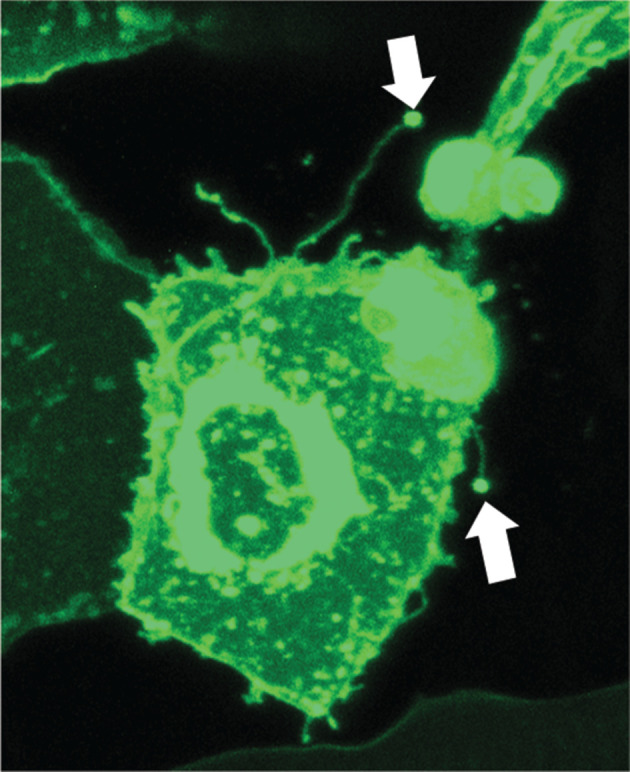
Keratinocytes extend airinemes. Arrows indicate airinemes extended from keratinocyte (*krt5+*, green) in zebrafish.

Intriguingly, airinemes are seen not only in pigment cells but also from several other cell types in zebrafish. For example, airineme-looking protrusions (with a vesicle at the tip) have been detected in keratinocytes (figure 4). Their cytoskeletal composition, dependence on macrophages and functional roles are under investigation. Such observations suggest airineme-mediated signalling could be used more in general, at least in zebrafish, and it is conceivable that airinemes in other organisms might be found. However, it remains to be determined in the future.

It would be interesting to explore whether airinemes are specialized in delivering Delta ligands or have the ability to deliver other signalling molecules similar to cytonemes. One of the speculations is whether the different types of signalling protrusions are optimized for delivering specific signalling molecules in different species and contexts. For example, airinemes deliver Delta ligand expressing vesicles to target cells in zebrafish; however, it has been shown that Delta can be transferred with cytonemes in *Drosophila* [30,36]. It is conceivable that larger numbers of Delta ligands can be transferred if they are packed into vesicles as compared to the thread-like connections, as seen in cytonemes, which lacks notable external vesicles [57]. Also, in zebrafish, Wnt ligand is delivered through cytonemes, and the ligands are located at the tip of cytonemes without vesicle-like structures [16]. Thus, it may be evolutionarily and/or functionally optimized for different levels of signalling requirements in different contexts and species.

## 6. Future perspectives

Collectively, the discoveries described above suggest that the mechanisms of signal propagation are much more complex than previously understood. Although the evidence for the functional importance of cellular protrusion-mediated signalling has been rapidly growing, it remains incompletely understood and its potential applications for human health-related problems remain largely unexploited. At present, we are only beginning to unravel this intercellular communication mechanism and do not yet know how general and prevalent it is in various biological systems. In this regard, key questions that need to be addressed are: (i) how do airinemes or other cellular projections distinguish between ‘correct’ target versus non-target cells? In other words, how are signalling specificity and directionality achieved? (ii) What other signalling molecules are inside the airineme vesicles? (iii) What are the molecular bases of airineme/macrophage-mediated signalling? Are there airineme-specific regulators? (iv) Do airinemes or other cellular projections exist and function in mammalian tissues *in vivo*, including humans? Importantly, since they transmit major signalling molecules, it is likely that their malfunction could be the origin of some human diseases, yet, at present, this is not recognized.

Additionally, to get a better view of the dynamic nature of airinemes or other cellular protrusion-mediated long-range signalling, it is essential to understand their cellular behaviours and signalling events at the tissue level, which is challenging to acquire systemic level of details with optical imaging. Since airineme extension is a temporal event and barely detectable with high-resolution confocal microscopy, scaling up the resolution into tissue level observation is challenging. Thus, it would be practical to approach this problem with mathematical modelling. It is expected that this would achieve a more systematic understanding and predictions of airineme-mediated signalling with interdisciplinary approaches.

Lastly, analysing the massive amount of imaging data with manual measures is not practical and potentially biased. Thus, it is crucial to develop methods to extract thin airineme or other cellular morphologies with real-time dynamics automatically by computational segmentation, followed by machine-learning-based optimization. Combining such techniques and computational modelling will enhance our understanding of cellular protrusion-mediated signalling in an unbiased and systematic manner in the future.

## Data accessibility

This article has no additional data.
